# From stillness to motion: 80 years after the first description of *Taenia solium* oncosphere hatching

**DOI:** 10.1186/1756-3305-7-12

**Published:** 2014-01-16

**Authors:** Fela Mendlovic, Adriana Garza-Rodríguez, Joaquin Carrillo-Farga, Fernando González-Domínguez, Pablo Maravilla, Ana Flisser

**Affiliations:** 1 Facultad de Medicina, Universidad Nacional Autónoma de México, México 04510 DF, Mexico; 2 Facultad de Ciencias de la Salud, Universidad Anáhuac del Norte, México Norte, Av. Universidad Anáhuac 46, Huixquilucan Estado de México 52786, Mexico; 3 Dirección de Investigación, Hospital General “Dr. Manuel Gea González” SSA, México 14000 DF, Mexico; 4 Instituto de Hematopatología, Tamaulipas 131, Col. Cuajimalpa, México 05000 DF, Mexico

**Keywords:** Activation, Cysticercosis, Hatching, Humans, Neurocysticercosis, Oncospheres, Pigs, *Taenia solium*, Video

## Abstract

**Background:**

Human neurocysticercosis (NCC) is a considered public health problem in many underdeveloped and developing countries. Because of the enormous increase in international tourism and migration, NCC nowadays is also found in some developed countries. Our group was the first to demonstrate that tapeworm carriers in the household are the main risk factor for acquiring cysticercosis in humans and pigs, since the disease results from the ingestion of microscopic tapeworm eggs.

**Findings:**

We had the opportunity to film the liberation of the embryo from the oncospheral membrane after the hatching of the egg, which is the activation process required for intestinal wall invasion by the onchosphere. Yoshino (J Formosa Med Ass **32**:139-142, 1933) described with great detail in diagrams and photographs this process eighty years ago after he infected himself with three living cysticerci in order to study the life cycle of *Taenia solium.* Other authors further described this process. Nevertheless it has never been filmed before. The purpose of this paper is to shift from stillness to motion since we can now show for the first time a movie of an activated oncosphere and its release from the oncospheral membrane.

**Conclusion:**

Oncospheral activation is the requisite for *T. solium* embryos to invade the intestinal mucosa and develop into cysticerci. This process has been amply described but here it is shown for the first time in motion; thus it may be of interest for readers of the journal and useful for educational purposes towards the control of NCC.

## Findings

The life cycle of *T. solium* includes the human being as the definitive host that harbors the intestinal tapeworm and the pig as the intermediate host in which the larval stage or cysticercus develops. Definitive hosts acquire the disease after ingesting undercooked infected pork meat, while cysticercosis is produced after swine consume infected human feces that contain tapeworm gravid proglottids full of eggs. In addition, accidental ingestion of these eggs can cause human neurocysticercosis (NCC), a public health problem in many developing countries and an emerging infectious disease in some developed ones [1-3]. NCC is a neglected tropical disease that is underfinanced and caused 25,341 disability adjusted life years (DALYs) in Mexico during 2005 due to epilepsy (90%) and severe chronic headaches (10%) associated to NCC [4]. Improvements in research, diagnosis, treatment and control have been achieved in Mexico, suggesting that NCC may no longer be a public health problem in this country [5]. This approach should be taken into consideration for the control of zoonoses and marginalized infectious diseases of poverty recently reviewed [6].

Our group was the first to demonstrate that patients with NCC are found mainly where a tapeworm carrier is part of the household [7], thus the egg is one of the main targets for the control of NCC. The morphology of tapeworm eggs has been described and illustrated in photographs and diagrams as early as 1933 by Yoshino [8], and afterwards by other authors [9-13]. Eggs measure between 26 and 34 μm and have a radial appearance because of the embryophore that surrounds and protects the oncosphere that is covered by the oncospheral membrane.

**Figure 1:**
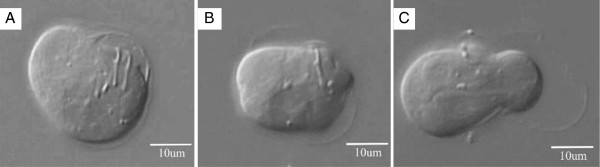
**Activation process of a *Taenia solium *oncosphere.** At the beginning of the activation process, an oncosphere completely surrounded by the oncospheral membrane is seen **(A)**. With time, the oncospheral membrane is detached and seen above and below the oncosphere **(B)**. At the end, the hexacanth is seen almost completely out of the oncospheral membrane, which remains, at the right side **(C)**. Hooks are evident in **(A)** and **(B)**.

After ingesting eggs, hexacanths must be released from their envelopes and penetrate the intestinal mucosa. Yoshino described the conditions for the hatching and activation of *T. solium* eggs after he infected himself with three live cysticerci to study the life cycle of *Taenia solium*. Disaggregation of the embryophore is known as hatching, while liberation from the oncospheral membrane is the activation process that take place in the presence of bile salts*.* Now, 80 years later, we had the opportunity to film the liberation of the hexacanth from the oncospheral membrane (film enclosed). By shifting from stillness to motion we show, for the first time, a movie filmed under an optic microscope (AX70 Olympus microscope with a Carl Zeiss camera attachment) of an oncosphere in the process of activation, as it liberates itself from the oncospheral membrane. Eggs were treated as described by Kyngdon *et al.*[13]. At the beginning of the film the oncosphere is seen after hatching, still surrounded by the oncospheral membrane, body movements are slow and increase in frequency as time elapses. Hooks also start moving and the structure of the hook described by Yoshino [8], as a stem with a terminal sickle-shaped portion, can be seen (Additional file 1: Movie S1 and Figure 1A and B). One minute after the beginning of the movie, a hook in the center of the embryo is seen seemingly scratching the inside of the oncospheral membrane. The oncosphere contracts and expands escaping from the membrane, probably through a hole, since a constriction around the embryo is evident. The hexacanth emerges towards the left side of the movie leaving the membrane behind (Additional file 1: Movie S1 and Figure 1C). The overall process of filming was speeded-up 5 times.

Oncospheral activation is the requisite for *T. solium* embryos to invade the intestinal mucosa and develop into cysticerci. This process has been amply described but here it is shown for the first time in motion, thus it may be of interest for readers of the journal and useful for educational purposes towards the control of NCC.

## Competing interests

The authors declare that they have no competing interests.

## Authors’ contributions

AGR and PM obtained and activated onchospheres. JCF, FGD, FM and AF participated in the filming process. FM and AF wrote the manuscript. All authors read and approved the final version of the manuscript.

## Supplementary Material

Additional file 1: Movie S1 The movie was filmed under an optic microscope and speeded-up 5 times. It shows the activation of a *Taenia solium* oncosphere. Click here for file
